# Global Prevalence of Restless Legs Syndrome and Its Associated Factors in Pregnant Women: A Systematic Review and Meta‐Analysis

**DOI:** 10.1002/hsr2.73165

**Published:** 2026-09-01

**Authors:** Nader Salari, Yassaman Khodayari, Roya Hassani, Fateme Gol Nazari, Masoud Mohammadi

**Affiliations:** ^1^ Department of Biostatistics, School of Health Kermanshah University of Medical Sciences Kermanshah Iran; ^2^ Medical Biology Research Center Kermanshah University of Medical Sciences Kermanshah Iran; ^3^ College of Business amd Law, Department of Organizational Studies University of the West of England Bristol UK; ^4^ Student Research Committee Kermanshah University of Medical Sciences Kermanshah Iran; ^5^ Research Center for Social Determinants of Health Jahrom University of Medical Sciences Jahrom Iran

**Keywords:** meta‐analysis, pregnancies, pregnancy, restless leg syndrome, systematic review

## Abstract

**Background and Aims:**

Restless leg syndrome (RLS) or Willis‐Ekbom's disease is a common neurosensory motor disorder, and pregnancy is one of the secondary factors that causes it. The purpose of this systematic review and meta‐analysis study is to obtain the percentage of prevalence of restless leg syndrome during pregnancy and to analyze the available evidence about this syndrome.

**Methods:**

The present study conducted the initial search in August 2022 and updated until December 2025. In this systematic review and meta‐analysis in order to find related studies, PubMed, Web of science, Science Direct, Scopus, Embase and Google scholar search engine using the keywords RLS Restless legs syndrome, Pregnancies, Pregnancy, and Gestation were searched. Random Effects Model was used to perform the analysis.

**Results:**

In the review of 19 studies with a sample size of 48,231 individuals, the meta‐analysis, the prevalence of restless legs syndrome in pregnant women was reported 17% (95% CI: 11.9–23.8). In examining the factors affecting the heterogeneity of studies and examining the effect of sample size on this heterogeneity, it was reported that with increasing sample size, the global prevalence of RLS in pregnant women decreased (*p* < 0.05).

**Conclusion:**

Our findings reveal that the prevalence of RLS in pregnant women is 17%. Considering the factors that affect this syndrome and also the effects that this syndrome has on the lives of pregnant women, particularly the quality of sleep and psychological issues, it is recommended to address this issue as a critical public health issue.

## Highlights

- In the review of 19 studies with a sample size of 48,231 individuals.
- The meta‐analysis, the prevalence of restless legs syndrome in pregnant women was reported 17%.
- It was reported that with increasing sample size, the global prevalence of restless leg syndrome in pregnant women decreased (*p* < 0.05).

**Abbreviations**
PRISMA: Preferred Reporting Items for Systematic Reviews and Meta‐Analysis
RLS: Restless legs syndrome
STROBE: Strengthening the reporting of observational studies in epidemiology for cross‐sectional study
WoS: Web of Science

## 1 Background

Restless legs syndrome (RLS) or Willis‐Ekbom's disease is a common sensorimotor neurodegenerative disorder with a prevalence of up to 10% in the general population. The disorder is characterized by an unpleasant crawling or crawling sensation in the legs or an irresistible urge to move the legs. The prevalence of RLS increases with increase of age and this disease is more common in women [1, 2, 3]. The prevalence of RLS based on IRLSSG diagnostic criteria in the general population is 15.3%–4% in the West [1, 2, 4, 5, 6, 7, 8, 9, 10]. It is also stated that it is less common among eastern or Asian populations [11].

RLS can be classified as primary or secondary based on the presence of associated conditions. The primary form is the most common type [12]. It has an innate pattern and affects patients who have no underlying disorder [12]. The primary form of RLS has a strong genetic background, and multiple genes are responsible; it appears from the age of 45 [1, 2, 12]. The secondary type is related to various underlying conditions and physiological changes such as pregnancy, iron deficiency, obesity, diabetes, and depression [1, 2, 12]. Also, Uremia and polyneuropathy, disruption of dopamine secretion in the brain, reduction of iron in the nervous system of end‐stage renal disease, and peripheral neuropathy are known as factors affecting secondary RLS [1, 2, 12].

Pregnancy is known as one of the factors influencing the occurrence of secondary restless legs syndrome [12]. Studies on the prevalence of RLS during pregnancy vary from 10.4% to 44% depending on the diagnostic criteria, trimester, and characteristics of the studied population [13].

The pathophysiology of RLS during pregnancy is not yet clear and may be related to iron or folate deficiency, high levels of estrogen, progesterone, and prolactin, psychomotor disorders, and radicular myopathy or peripheral neuropathy [14, 15, 16, 17, 18]. However, in a series of studies, the relationship between restless legs syndrome in pregnancy with factors such as older pregnancy, low socio‐economic status, higher body mass index, weight gain during pregnancy, low physical activity, smoking and alcohol consumption, as well as between low iron reserves, low hemoglobin level Hypochromic anemia of pregnancy blood pressure, chronic blood pressure, high blood pressure and preeclampsia caused by pregnancy have been identified [3, 15, 19, 20, 21, 22, 23, 24, 25, 26]. Pregnant women with restless legs syndrome complain of its adverse effects on sleep quality, insomnia, sleep disturbance, cognitive functions, mood, and quality of life due to sleep disturbance [27, 28].

According to the explanations provided and the significance of this issue among pregnant women, this study will investigate the global prevalence of restless legs syndrome in pregnant women and the factors affecting it.

## 2 Methods

The present study did the initial search in August 2022. In this systematic review and meta‐analysis in order to find related studies, PubMed, Web of Science, Science Direct, Scopus, Embase, and Google Scholar search engine using the keywords RLS, Restless legs syndrome, Pregnancies, Pregnancy were searched. The identified information was transferred to the Information Management Software, EndNote. In order to maximize the number of relevant studies, the list of references used in the identified related articles was reviewed manually. Besides, the searches were last updated in December 2025. The databases were systematically searched using defined terms. Boolean operators (“AND” and “OR”) were applied to combine keywords and maximize the sensitivity and comprehensiveness of the search strategy. In addition, Medical Subject Headings (MeSH) were used via the MeSH browser to identify standard terms.

PubMed search sample: (((((((Restless legs syndrome[Title/Abstract]) OR (Restless Legs[Title/Abstract])) OR (Willis Ekbom Disease[Title/Abstract])) OR (Willis Ekbom Syndrome[Title/Abstract])) OR (Wittmaack‐Ekbom Syndrome[Title/Abstract])) OR (RLS[Title/Abstract])) AND (Pregnancy[Title/Abstract])) OR (Pregnant People[Title/Abstract])) OR (Prenatal Care[Title/Abstract])) OR (Pseudopregnancy[Title/Abstract])) OR (Pregnancies[Title/Abstract])))))))

### 2.1 Study Inclusion Criteria

Studies that reported the prevalence of RLS in pregnancy, studies that their full text was available, studies that provided sufficient data (sample size, prevalence percentage), and observational studies (cross‐sectional, case‐control, and cohort).

### 2.2 Study Exclusion Criteria

Case series, review studies, repetitive studies, and studies with insufficient data (failure to provide sample size, inadequate follow‐up period from the onset of symptoms, and failure to provide prevalence percentage).

### 2.3 Study Selection

EndNote software was used to organize the studies. Initially, the studies that were repeated in different databases were excluded from this study. In the initial evaluation, the titles and abstracts of the articles were carefully evaluated and articles unrelated to the purpose of the research were removed. In the second stage (the secondary evaluation of the studies), the full text of the related articles was examined and the studies that met the conditions of the inclusion in the study were included. In order to increase reliability and avoid bias, initial screening was conducted by two researchers to determine whether the eligibility criteria were met. In cases where there was a disagreement between the two researchers, with the participation of a third researcher, and reaching a consensus, the article would be finalized. Nineteen studies entered the third stage, which was the quality evaluation.

### 2.4 Quality Evaluation

In order to validate and evaluate the quality of articles, a checklist was used according to observational studies. The Strengthening the Reporting of Observational Studies in Epidemiology (STROBE) checklist consists of six scales including: title, abstract, introduction, methods, results, and discussion. In total, this instruction consists of 32 items. These 32 items include different methodological aspects of the study, including the title, statement of the problem, study objectives, type of study, statistical population of the study, sampling method, determining the appropriate sample size, definition of variables and procedures, study data collection tools, statistical analysis methods, and findings. Based on this, articles with a score of 16 and above were considered to be good and moderate in terms of methodological quality, and articles with a score below 16 were considered to be poor in terms of methodological quality and therefore excluded from the study.

### 2.5 Data Extraction

Data extraction was conducted by two researchers using a previously prepared checklist. This checklist included: author's name, publication year, research location, sample size, gender and age of participants, number of chronic fatigue sufferers, and prevalence percentage.

### 2.6 Statistical Analysis

The I^2^ test was used to evaluate the heterogeneity of the selected studies. In order to check the publication bias, due to the large volume of samples included in the study, the Egger test was used at a significance level of 0.05, as well as the corresponding Funnel Plot. Meta‐regression was also performed on factors affecting heterogeneity based on sample size and year of study. Data analysis was conducted using Comprehensive Meta‐Analysis Version 2.0 software.

## 3 Results

**Figure 1:**
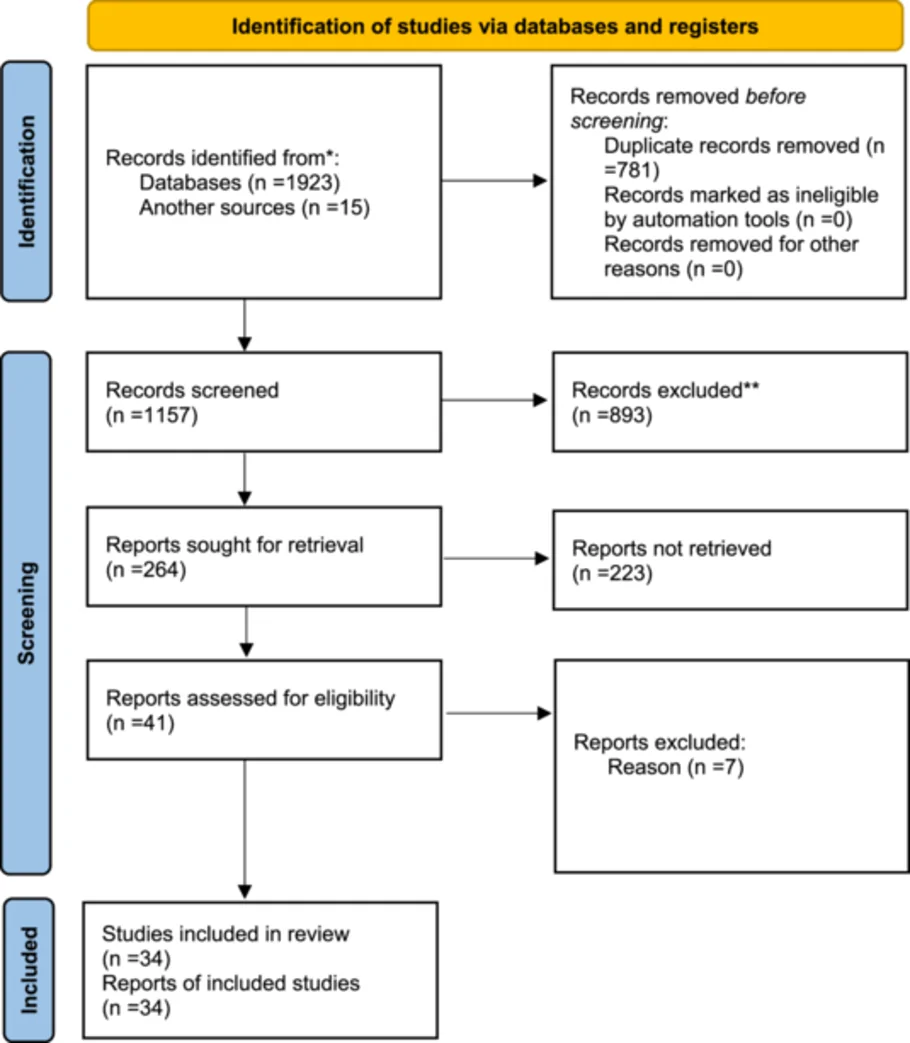
PRISMA 2020 chart based on initial search, screening, and identification of final articles.

In this systematic review and meta‐analysis of studies, the global prevalence of restless legs syndrome in pregnancy was evaluated. Without any time, limit until December 2025 and based on PRISMA guidelines 2020. 1923 possible related articles (PubMed:233, Science Direct:106, Embase:391, Web of Science:374, Google Scholar:155, Scopus:664) were identified and transferred to the Information Management Software EndNote, and after removing duplicates, irrelevant, and low‐quality articles finally, 34 were included in the final analysis. The reviewed studies were based on the four‐step process of PRISMA 2020, including identifying articles, screening, checking the acceptance criteria of articles, and finally, the articles included in the meta‐analysis were reviewed (Figure 1). Finally, 32 studies were included in the final analysis, their information is listed in Table 1. From the final number of 34 articles obtained, 19 cross‐sectional studies were used to investigate the prevalence meta‐analysis, and 14 observational studies, including cohort and case‐control studies, and one cross‐sectional study not reported in Table 1, along with 10 cross‐sectional studies reported in Table 1, were used to investigate the associated factors in Table 2.

**Table 1:** Information extracted from the final reviewed studies.

Author et al.	Year	Region	Study design	Age	Population	Prevalence%
M. Vahdat et al. [29]	2011	Iran	Cross sectional	26.6	433	17.8
R. Sikandar et al. [30]	2005	Pakistan	Cross sectional	30 years or above	271	30
X. Shang et al. [31]	2014	China	Cross sectional	26	1584	11.2
T. Tunc et al. [32]	2007	Turkey	Cross sectional	24.81	146	26.02
L. Č. Jurjević et al. [33]	2017	Croatia	Cross sectional	18–50	384	7.1
G. Esposito et al. [34]	2017	Italy	Cross sectional	35	648	20.4
S. Harano et al. [35]	2008	Japan	Cross sectional	29.9	20,781	2.9
S. Ismailgullar et al. [36]	2008	Turkey	Cross sectional	< 20–39	983	10.48
M. Minár et al. [37]	2014	Bratislava	Cross sectional	30.81	300	31.33
D. O. Oyieng'o et al. [38]	2016	Rhode Island	Cross sectional	29	1000	35.5
D. A. Galdino Alves et al. [39]	2010	Brazil	Cross sectional	27.3	524	13.5
P. Abedi et al. [40]	2018	Iran	Cross sectional	27.5	700	28.9
A. S. Al Shidhani et al. [41]	2022	Oman	Cross sectional	29.8	305	15.7
K. Suzuki et al. [42]	2003	Japan	Cross sectional	29.3	17,270	19.9
S. Panvatvanich et al. [43]	2018	Thailand	Cross sectional	28.6	214	11.2
S. Miri et al. [44]	2014	Iran	Cross sectional	30.6	443	17.8
M. Khan et al. [45]	2018	Saudi Arabian	Cross sectional	30.11	517	21.3
I. Hudić et al. [46]	2020	Bosnia & Herzegovina	Cross sectional	25.7	1228	22.7
B. Çakmak et al. [47]	2014	Turkey	Cross sectional	27	500	15.4

**Table 2:** Report of factors affecting restless leg syndrome in pregnant women based on studies reviewed in a systematic review.

Author & Year	Location	Type of the study	Results	
			*p*‐valve	Evaluated risk factor
A.S. Almeneessier et al., 2019 [48]	Saudi Arabia	Case‐control	0.743	Age
			< 0.001	BMI
			< 0.001	Diabetes mellitus
			< 0.001	Vitamin D deficiency
			< 0.001	Anemia (hemoglobin less than 11)
M.B. Fawale et al., 2017 [49]	Nigeria	Case‐control	0.003	Age
			0.018	Monthly Income
			0.019	Education
			< 0.001	Ethnicity
			< 0.001	Occupation
			0.000	Cigarette
			0.000	Alcohol
			0.273	Coffee
			0.030	High Blood Pressure
			0.000	Diabetes
			0.493	Chronic kidney disease
			0.000	Sleep Problems
			< 0.001	Normal sleep duration
			< 0.001	menarche (first menstruation)
			0.000	Number of pregnancies
				Adverse pregnancy outcomes in the past:
			0.216	leg cramps
			0.824	leg swelling
			0.036	Foot edema
			0.016	Systolic blood pressure
			< 0.001	diastolic blood pressure
			< 0.001	proteinuria
			< 0.001	Hemoglobin concentration
			0.061	RLS Symptoms
			0.499	RLS History
E. Yildirim et al., 2020 [50]	Turkey	Case‐control	0.146	Platelet to lymphocyte ratio
			0.310	lymphocyte
			0.485	Neutrophil to lymphocyte ratio
			0.06	Age
			0.120	BMI
			0.718	Number of pregnancies
			0.90	number of births
			0.90	Number of live pregnancies
			0.016	Abortion
			0.368	Maternal age by week
			0.001	Biparietal diameter of the fetus
			0.048	Fetal femur bone length
			0.085	The size of the fetal abdomen
			0.30	Amniotic fluid index
			0.072	hemoglobin
			0.332	Average cell volume
			0.132	The number of white blood cells
			0.139	Platelet count
			0.04	Neutrophil count
			0.743	Age of Pregnancy
A. Hatanaka et al., 2017 [51]	Japan	Case‐control	0.766	Age
			0.061	number of births
			0.854	hemoglobin
			0.825	Iron
			0.516	ferritin
			0.087	The duration of childbirth
			0.053	Blood loss
			0.001	Type of delivery
			0.001	Labor induction
			0.736	An increase in the number of births
			0.111	Postpartum contractions
			0.317	Age of Mother
Vahed et al., [29] (2016)	Iran	Case‐control	0.002	BMI
			0.654	Mother's Occupation
			0.118	number of births
			0.025	Pregnancy Age
			0.032	Hemoglobin of the second trimester
			*< *0.001	Sleep Quality
			0.081	Age
Sh. Ma et al., 2015 [52]	China	Case‐control	0.168	BMI
			0.552	Number of pregnancies equal to or greater than 2
			0.413	The number of births is equal to or more than 2
			*< *0.001	High blood pressure caused by pregnancy
			0.070	Primary blood pressure in pregnancy
			*< *0.001	Daily intake of iron supplements
			0.084	Daily intake of folate supplements
			0.854	Age
A. Neyal et al., 2015 [53]	Turkey	Case‐control	0.442	Age Group
			0.630	Education
			0.307	Current or previous smoking history
			0.073	Use of iron and supplements
			0.803	Current BMI
			0.825	Age
I. Ozer et al., 2016 [54]	Ankara (Turkey)	Cohort (prospective)	0.188	Number of Pregnancies
			0.176	number of births
			*< *0.001	Education Level
			*< *0.001	Financial Status
			*< *0.001	Family Type
			*< * 0.001	Thyroid disease
			*< *0.001	Iron Consumption
			*< *0.001	Calcium Consumption
			*< *0.001	Magnesium Consumption
			*< *0.001	Hemoglobin level
			*< *0.001	Hemoglobin less than 11
			0.340	Age
D. Devaraj et al., 2019 [55]	India	Case‐control	0.070	Socio‐economic Status
			0.8	BMI
			0.6	Systolic blood pressure
			0.8	Age of Pregnancy
			0.3	iron supplement
			0.03	Sleep
			0.7	hemoglobin
			0.3	ferritin
			0.2	Child's Weight Upon Birth
			*< *0.0001	Age
M. Na et al., 2020 [56]	USA	Cohort (prospective)	*< *0.0001	Education
			*< *0.0001	Number of pregnancies
			*< *0.0001	Annual Income
			*< *0.0001	Nutrition status before pregnancy (height and weight)
			*< *0.0001	BMI
			*< *0.0001	Nutritional status during pregnancy
			*< *0.0001	Iron deficiency anemia
			0.336	Child's Gender (male)
P.H. Chen et al., 2012 [21]	Taiwan	Prospective	0.774	Age
			0.963	BMI
			0.113	Level of Education
			0.219	Number of Pregnancies
			0.325	number of births
			0.987	Exercise
			0.789	Cigarette Consumption Prior to Pregnancy
			0.068	Alcohol Consumption Prior to Pregnancy
			0.126	Coffee Consumption Prior to Pregnancy
			0.013	folic acid
			0.006	Iron
			*< *0.001	Anemia
			0.040	Thyroid disease
			0.684	hepatitis
			0.614	Diabetes
			0.001	Ulcers
G.L. Dunietz et al., 2017 [57]	USA	Cohort	0.9	Age
			0.04	Ethnic Background
			0.03	Education
			0.1	Pre‐pregnancy BMI
			0.1	Wight Increase During Pregnancy
			0.2	Age of Pregnancy
			0.02	Zero parity
			*< *0.001	Epworth Sleep Scale
			0.9	Having high blood pressure
			0.1	The presence of diabetes
			*< *0.001	Low Sleep Quality
			*< *0.001	Poor performance during the day
			*< *0.001	Normal Snoring
			0.000	Number of RLS (frequency)
T.W. Goecke et al., 2020 [58]	Germany	Prospective	0.05	Age
			0.05	Marital Status
			0.05	Number of Children
			0.05	Education
			—	Characteristics related to pregnancy:
			0.000	Type of delivery
			0.05	Moving the baby after delivery
				Complications related to pregnancy:
			0.05	Preeclampsia
			0.05	eclampsia
			0.05	Hellp syndrome
			0.054	Gestational Diabetes
			0.05	Hypothyroidism
			0.064	High Blood Pressure
			0.05	Anemia after childbirth
			0.05	Duration of Pregnancy
			0.05	Weight of Child Upon Birth
J. Ph. Neau et al., 2010 [59]	France	Prospective	0.004	Age
			0.19	Singlehood
			*< *0.0001	3rd Trimester
			0.07	Zero parity
			0.007	Having problems in pregnancy
			0.086	Iron Consumption
			0.01	Magnesium Consumption
			0.47	Vitamin Consumption
			*< *0.0001	Sleeping Pills Consumption
			0.58	Anti Depression Factor
			0.015	Homeopathy Treatment
T. Tune 2007 [32]	Turkey	Cross‐sectional	0.244	age
			0.260	Pregnancy week
			0.787	Other medical issues
			0.004	parity
			0.243	Having problems in pregnancy
			0.012	Medication consumption, iron
			0.306	Sleep problems
			0.002	Excessive sleepiness during the day
			0.007	Leg cramps
M. Khan et al., 2018 [45]	Saudi Arabia (Riadh)	Cross‐sectional	0.947	Age
			0.573	Education level
				Co‐morbidities and potential risk factors:
			0.421	Coffee Consumption
			0.195	Tea Consumption
			0.012	3rd trimester of pregnancy
			0.757	Obesity
			0.917	Abortion
			0.813	Diabetes Mellitus
			0.139	High blood pressure
			0.332	Depression
			0.065	Asthma
			0.441	Number of pregnancies
			0.260	number of births
				Symptoms of sleep disorder
			0.046	Excessive sleepiness during the day
			0.001	Insomnia
			0.002	Pittsburgh Sleep Quality Index
Abedi et al., 2018 [40]	Iran	Cross‐sectional	0.7	Age
			0.03	BMI
			0.004	Pregnancy age
			< 0.001	parity
			0.02	Pregnancy trimester
			0.390	Occupation
			0.17	Economic situation
			< 0.001	Family history of RLS
			< 0.001	The history of RLS during previous pregnancy
			< 0.001	Caffeine Consumption
			0.33	Taking iron supplements during pregnancy
Miri et al., 2014 [44]	Iran	Cross‐sectional	0.016	Age
			0.099	zero parity
			0.342	Family history of RLS
			0.0001	Duration of RLS (months)
			0.789	RLS severity index
			0.524	Sleep delay
			0.046	Sleep duration
			0.133	Preterm delivery
			0.700	Pregnancy duration (weeks)
			0.213	Daily sleepiness
			0.372	Early wake up
			0.233	Sleeplessness
			0.408	Type of delivery (natural or cesarean section)
S. Ismailogullari et al., 2010 [36]	Turkey	Cross‐sectional	0.053	Age
			0.788	Education
			0.685	Number of previous pregnancies
			0.830	Income level
			1.000	Occupation
			0.930	Number of children
			0.037	Treatment with iron and folate
P. Lolekha et al., 2018 [60]	Thailand	Cross‐sectional	0.42	Age
			0.75	Pre‐pregnancy BMI
			0.71	BMI in the 3rd trimester
			0.02	hemoglobin
			0.13	hematocrit
			0.76	MCV
			0.89	Multigravida
			0.98	Multiparity
			0.48	History of abortion
			0.90	Diabetes mellitus
			< 0.001	Thyroid disease
			0.37	Blood pressure
			> 0.01	Anemia (hemoglobin equal to and less than 11 grams per deciliter)
			0.62	Smoking history (cigarettes)
			0.53	History of alcohol Consumption
			0.52	History of caffeine Consumption
			> 0.01	History of RLS
			0.58	Complications of childbirth
			0.22	Female child
			0.58	Preterm delivery
			0.77	Cesarean section
			0.35	Apgar score
			0.26	Weight upon birth
A.S. Al shidhani et al., 2022 [41]	Oman	Cross‐sectional	0.101	Age
			0.154	BMI
			0.044	Family history of RLS
			0.143	Number of pregnancies
			0.127	number of births
			0.516	Education level
			< 0.001	Personal history of RLS
			0.092	Severe nausea and vomiting of pregnancy
			0.409	Previous history of diabetes mellitus/gestational diabetes
			0.208	History of high blood pressure
			0.686	Hemoglobin level
			0.229	Maternal age at delivery
			0.315	Weight upon birth
			0.312	Type of delivery (natural or cesarean section)
M. Minár et al., 2013 [61]	Slovakia	Cross‐sectional	0.94	Age
			0.89	BMI
			0.06	Duration of pregnancy
			0.63	Number of pregnancies
			0.19	Child's wight upon birth
			< 0.001	Weight gain during pregnancy
X. Shang et al., 2014 [31]	China	Cross‐sectional	0.625	Age
			0.324	BMI
			0.145	community (urban or rural)
			0.622	Pregnancy
			0.084	Family history
			0.088	Smoking before pregnancy
			0.075	Alcohol consumption before pregnancy
			0.593	Tea consumption before pregnancy
			0.017	Folic Acid
			0.004	Iron
			0.013	Anemia
			0.014	Thyroid disease
			0.192	Diabetes
			0.24	varicose veins
			0.04	Arthritis (joint inflammation)
			< 0.001	RLS history in the previous pregnancy
R. Sikandar et al., 2005 [30]	Pakistan	Cross‐sectional	< 0.001	When the pregnant woman had no history of RLS
			< 0.001	Family history of RLS
			0.09	First pregnancy
			0.06	Abortion
			0.09	Hemoglobin equal to and less than 11 g/dL
			0.09	Age of 30 or more
			0.67	iron therapy
			0.41	Folate therapy
			0.41	Folate therapy
B. Cakmak et al., 2014 [47]	Turkey	Cross‐sectional	0.706	Age
			0.608	Number of pregnancies
			0.785	number of births
			0.650	Abortion
			0.04	Pregnancy age (weeks)
			0.003	Weight
			0.7	Height
			0.037	BMI
			0.009	Iron supplement
			0.207	Multivitamin supplement
			0.529	Diabetes
			0.433	Blood pressure
			0.694	Thyroid dysfunction
			0.4	hemoglobin
			0.375	hematocrit
			0.063	TSH
			0.105	T4
			0.329	BUN
			0.370	Creatinine
			0.621	AST
			0.796	ALT

**Figure 2:**
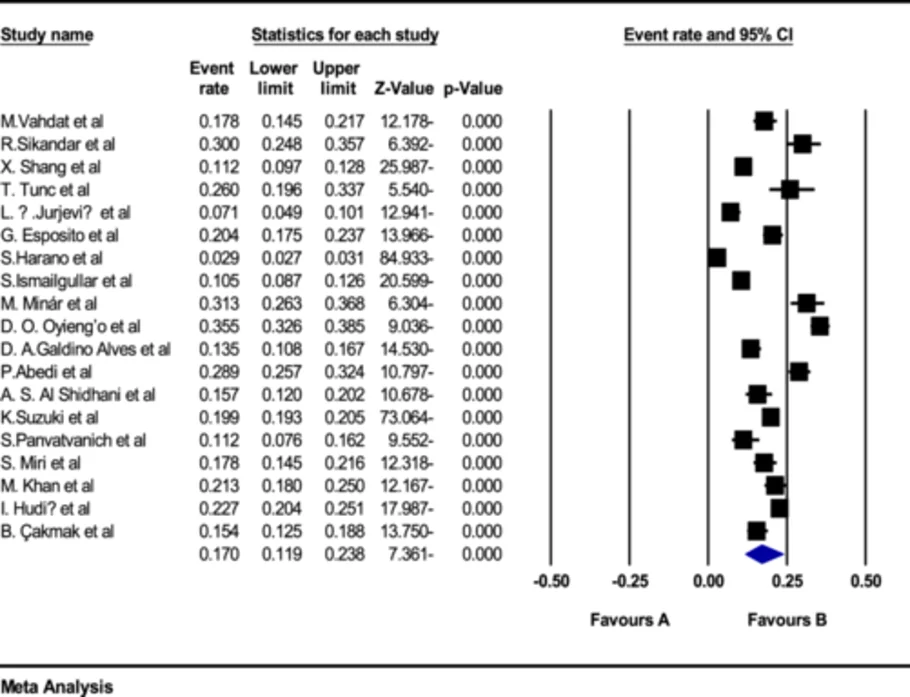
Forest Plot of global prevalence of RLS among pregnant women based on the random effects method.

**Figure 3:**
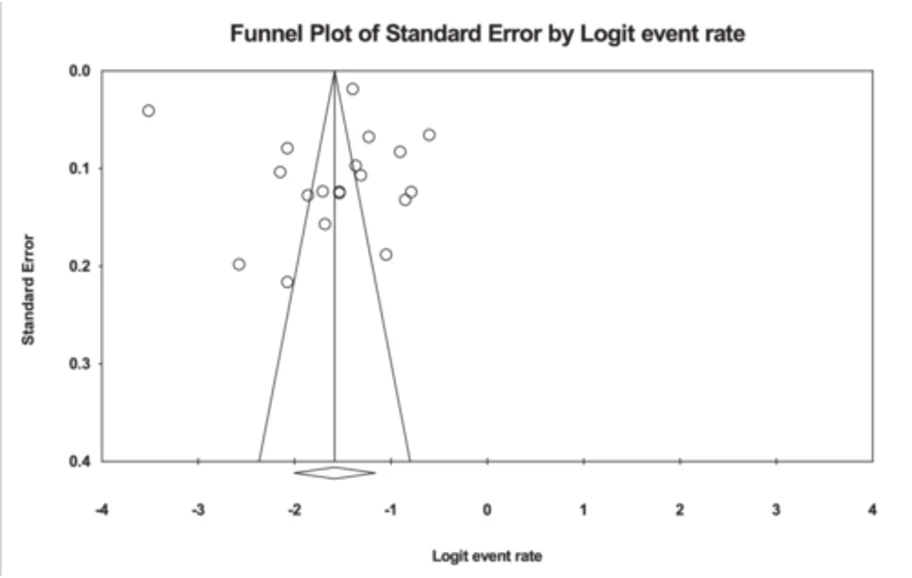
Funnel plot of the publication bias in the reviewed studies.

In the review of 19 studies with a sample size of 48,231 individuals, the I^2^ heterogeneity test revealed a high level of heterogeneity (I^2^ = 99.3). Subsequently, based on this, the Random Effects Method was used to analyze the results. Hence, based on the meta‐analysis, the global prevalence of RLS was reported in pregnant women as 17 (95% CI: 11.9–23.8) (Figure 2), and the Egger test demonstrates the absence of publication bias in studies based on the results of the statistical test, although given the high heterogeneity in prevalence studies, this absence of bias is not very proven (*p* = 0.984; Figure 3).

**Figure 4:**
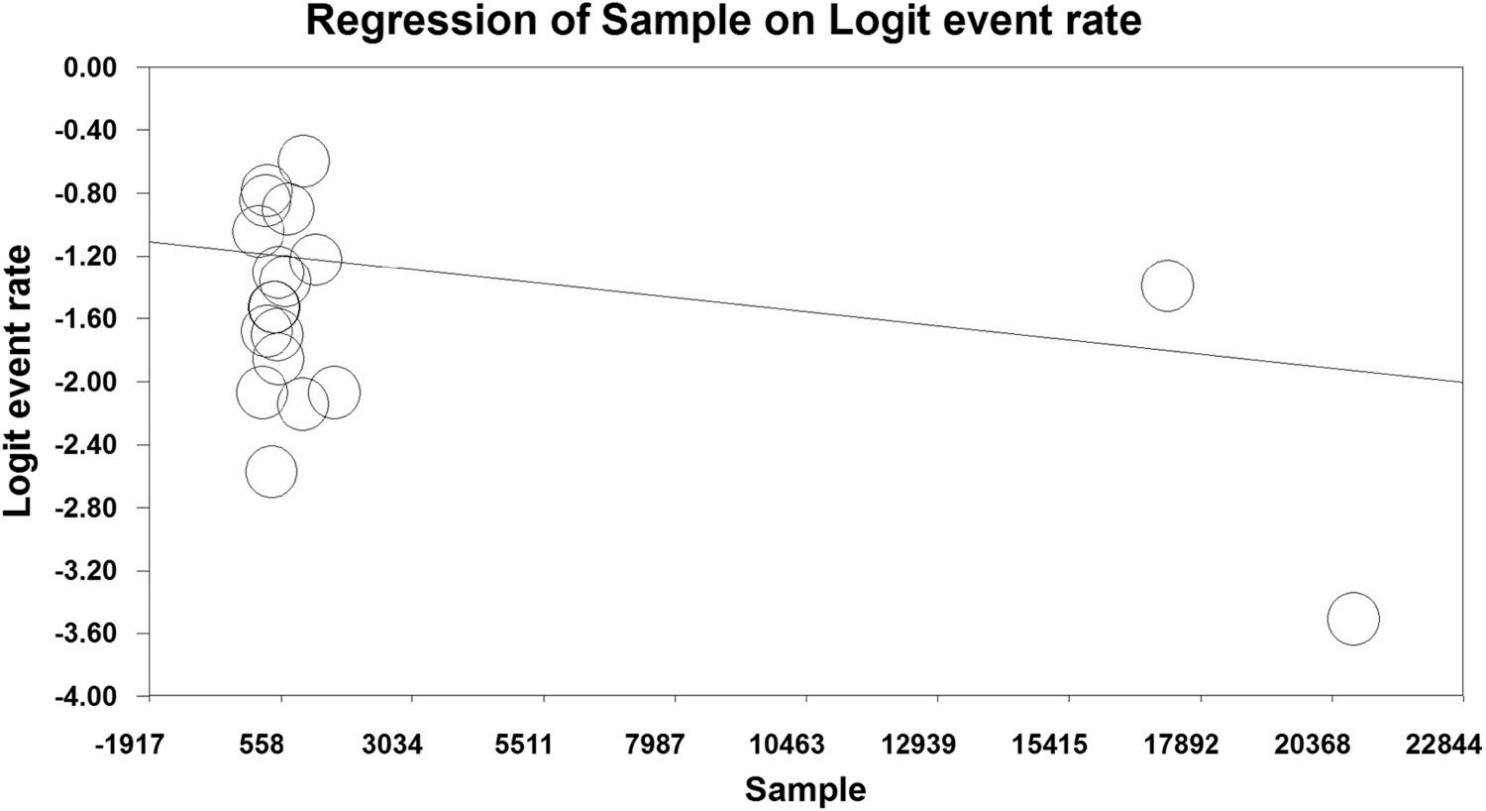
Met regression of the effect of sample size on the global prevalence of restless leg syndrome in pregnant women.

**Figure 5:**
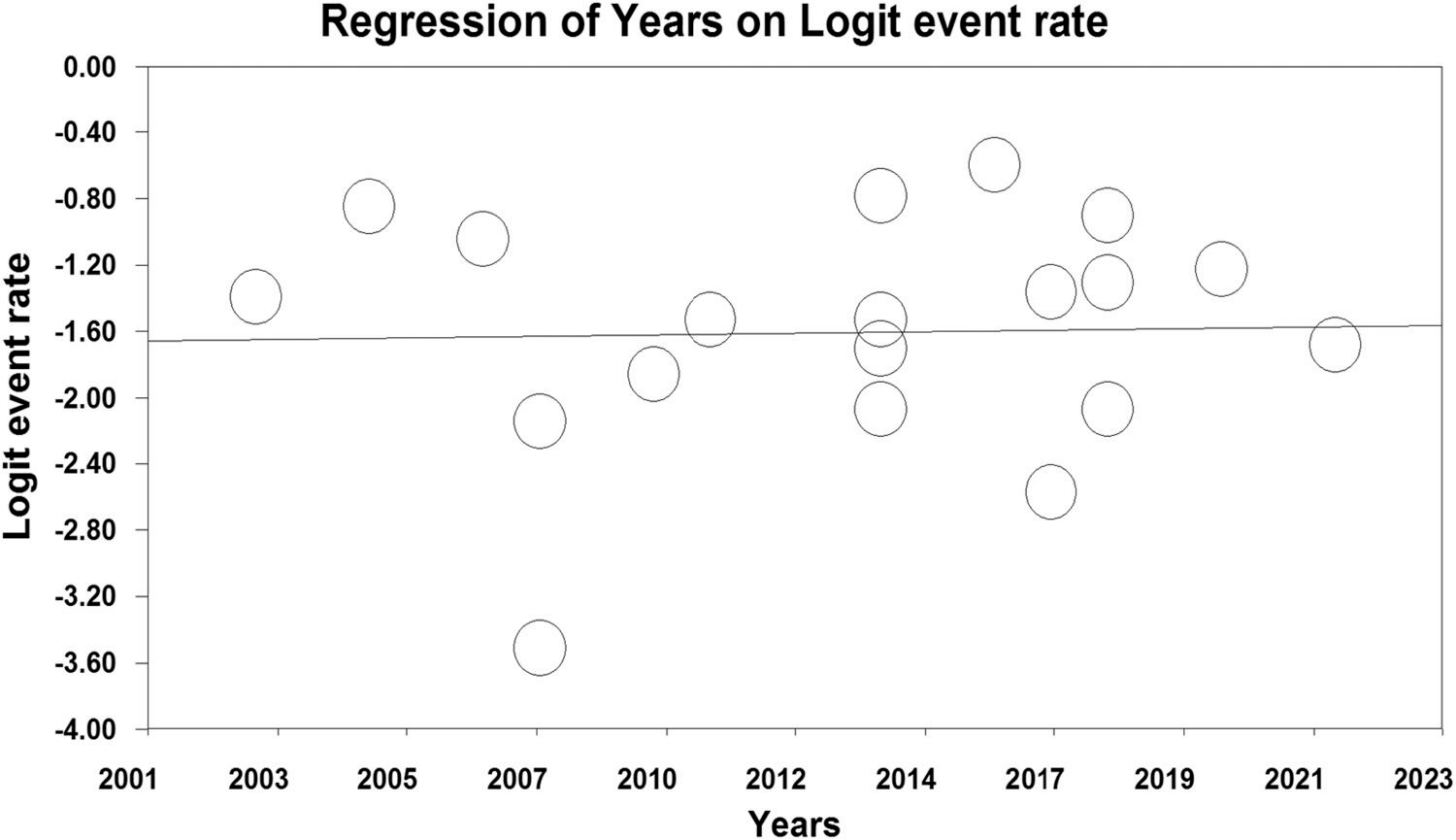
Meta‐regression of the effect of the study year on the global prevalence of restless leg syndrome in pregnant women.

In examining the factors affecting the heterogeneity of studies and examining the effect of sample size on this heterogeneity, it was reported that with increasing sample size, the global prevalence of restless legs syndrome in pregnant women decreased (*p* < 0.05) (Figure 4). In addition, it was reported that globally, restless legs syndrome in pregnant women increased with increasing year of study, which results were not statistically significant based on the test (*p* = 0.101; Figure 5).

In the present study, to find the risk factors with the highest level of significance, we examined the studies. The results of each of the studies are as below; In the study of A.S. Almeneessier et al. [48] BMI, diabetes mellitus and vitamin D deficiency, in the study of M.B. Fawale et al. [49] age, monthly income, education, ethnicity, occupation, smoking, alcohol, high blood pressure, diabetes, chronic kidney disease, sleep problems, normal sleep duration at night, menarche (first menstruation), number of pregnancies and also the following previous pregnancy problems It has been investigated that leg edema, systolic blood pressure, diastolic blood pressure, hemoglobin concentration and proteinuria have been reported to be related.

In the study of A. Hatanaka et al. [51], the type of delivery and induction of labor; in the study of Vahad et al. [29], Body Mass Index, maternal age, second trimester hemoglobin and sleep quality; in the study of Sh. Ma et al. [52] daily consumption of iron supplementation and high blood pressure caused by pregnancy, in the study of I. Ozer et al. [54] education level, economic status, family type, thyroid disease, iron consumption, calcium consumption, magnesium consumption, hemoglobin level, and hemoglobin less than 11.

In the study of M Na et al. [56] age, number of pregnancies, education, income (annual), nutritional status before pregnancy (height and weight), nutritional status during pregnancy, BMI and iron deficiency anemia, in the study of P.H. Chen et al. [21] Folic acid, iron, anemia, thyroid disease and peptic ulcer, in the study of G.L. Dunietz et al. [57] race/ethnicity, education, nulliparity, Epworth sleep scale, poor sleep quality, poor daytime performance, habitual snoring and frequency or frequency) RLS, in the study of T.W. Goecke et al. [58], the only factor is the type of delivery.

In the study of J. Ph. Neau et al. [59] age, third trimester, presence of pregnancy problems, magnesium intake, vitamin intake, antidepressant agent and homeopathic treatment, in the study of T. Tune et al. [32], parity, iron and drug use, the presence of excessive sleepiness during the day, and the presence of leg cramps, in the study of M. Khan et al. [45], the name and the third trimester of pregnancy and also, among the symptoms of sleep disorders, excessive sleepiness during the day, insomnia and Pittsburgh sleep quality index.

In the study of Abedi et al. [40] BMI, maternal age, parity, trimester of pregnancy, family history of RLS, history of RLS in Previous pregnancy and caffeine consumption, in Miri et al.‘s study [44], age and duration of RLS (in months), in S. Ismailogullari et al.‘s study [36], the only treatment factor with folate, in P. Lolekha et al.‘s study [43]) Hemoglobin, thyroid disease, anemia (hemoglobin equal to and less than 11 g/dL) and previous history of RLS, A.S. Al shidhani et al.‘s study [41] Family history of RLS and personal history of RLS.

In M. Minar's study et al. [61], the only factor of weight during pregnancy, in the study of X. Shang et al. [31], folic acid, iron, anemia, thyroid disease, varicose veins, and arthritis (arthritis), in the study of R. Sikandar et al. Previous pregnancy and history of RLS in the past (when the person was not pregnant) and in the study of B. Cakmak et al. [47], four factors of maternal age based on weeks, weight, BMI, and iron supplementation were identified as factors with the highest level of significance (Table 2).

## 4 Discussion

Restless legs syndrome (RLS) is a sleep‐related movement disorder that causes a strong, nearly irresistible urge to move your legs. Symptoms are usually worse when you're at rest in the evening or at night [1, 2, 3]. Although, RLS is common in the general population, its prevalence is higher among pregnant women, and pregnancy is also a common risk factor for the secondary RLS [1, 2, 13].

The relationship between pregnancy and RLS is well known, although there are few studies that have investigated it in detail [62]. In the studies conducted, the prevalence of this syndrome in pregnancy has been reported between 11% and 27% [20, 63]. The highest rate (35.5%) was reported by Oyieng'o DO et al. [38, 61]. Based on the studies and the reported statistics, it can be concluded that the prevalence of RLS in pregnant women is higher in the third trimester of pregnancy [64, 65, 66, 67, 68, 69]. In a study conducted by Shang et al., the prevalence rate in the surveyed population was reported to be 6% in the first quarter, which reached 17.2% in the third quarter, this confirms the stated content [64, 65, 66, 67, 68, 69, 70].

Hormonal changes during pregnancy have been mentioned as factors influencing restless legs syndrome in this period, since the levels of estrogen, prolactin, and progesterone increase, especially in the third trimester of pregnancy [13, 20, 23]. It has also been mentioned that these hormonal changes increase in the third trimester, which causes the symptoms of this syndrome in affected people [19]. Dopamine has also been mentioned as a hormone regulator, which has a definite regulatory effect, especially on the level of prolactin [13, 20, 68, 69].

Among the other factors affecting the prevalence of RLS during pregnancy, it can be mentioned that the prevalence differs in various geographical areas, this has been reported in a series of studies that in Western countries with a rate of 5.5–14.3 which is more than Asian countries with a rate of 1–3.9. The cause of this phenomenon is the genetic differences in people in different geographical areas [1, 10, 71, 72, 73, 74, 75]. However, in the study conducted by Sunil Rangarajan and his colleagues, no difference was observed between Brazilian ethnic populations and other investigated populations.

Several social and individual factors have also been investigated in studies, which can be mentioned as smoking. This has been known to be effective in several studies with the prevalence of restless legs syndrome, both in pregnant women and the general population. Although, it has been mentioned that if smoking has been used as a factor to reduce pressure and psychological stress, it acts as a confusing factor and may reduce the prevalence and have an effect as a modifying factor. Therefore, it should be considered in addition to the mental factors, yet psychological problems should also be examined [7, 13, 76, 77]. Also, in another study, no relationship between smoking and the prevalence of RLS was detected, and the effect of this factor was rejected [78]. Also, the number of parties was investigated, and no definite conclusion was reached about it; hence, in a study by Berger and his colleagues, it is mentioned that the number of children born is related to the prevalence and severity of restless legs syndrome in pregnancy [7]. However, in several studies, it is mentioned that the prevalence of this syndrome in primiparous women is no different than multi‐gravid individuals [13, 64].

An important complication of restless legs syndrome in pregnant women is the effect of this syndrome on sleep quality and sleep duration, which has been stated that the average sleep duration among pregnant women with RLS (7.40 h; SD, 1.53) compared to women without RLS (7.53 h; SD, 1.33). This is significantly shorter and also has an effect on the degree of effectiveness and relaxation of sleep [27, 28, 79], given the fact that pregnant women could have paresthesia symptoms and symptoms related to joint stiffness. This causes lumbar‐pelvic and sciatica pain and vein dilatation, which is a characteristic of pregnancy, and with a possible effect on venous return, it helps in the occurrence of muscle cramps [80, 81]. The effect of this syndrome on the quality of sleep and sleep duration of pregnant women causes fatigue and sleepiness during the day; if it becomes severe, it can have wide social and economic consequences [79].

## 5 Conclusion

Our findings show that the prevalence of restless legs syndrome in pregnant women is 17%. The syndrome has a significant impact on pregnant women's sleep problems, and it increases their mental and emotional problems, as well as having the subsequent impact on social and economic aspects. Hence, it is recommended that extensive investigations to be carried out in this field. Also, in the future research, the impact of mental factors and stress could be considered and investigated, and measures should be taken to reduce their adverse effects as an important public health issue.

## Author Contributions

**Nader Salari:** conceptualization, writing – original draft. **Yassaman Khodayari:** investigation, writing – original draft. **Roya Hassani:** writing – review and editing, supervision. **Fateme Gol Nazari:** investigation, writing – original draft, writing – review and editing. **Masoud Mohammadi:** conceptualization, investigation, writing – original draft, writing – review and editing, methodology, software, project administration, supervision.

## Funding

The authors have nothing to report.

## Ethics Statement

The authors have nothing to report.

## Consent

The authors have nothing to report.

## Conflicts of Interest

The authors declare no conflicts of interest.

## 1 Transparency Statement

The Corresponding author Masoud Mohammadi affirms that this manuscript is an honest, accurate, and transparent account of the study being reported; that no important aspects of the study have been omitted; and that any discrepancies from the study as planned (and, if relevant, registered) have been explained. All authors have read and approved the final version of the manuscript [CORRESPONDING AUTHOR] had full access to all of the data in this study and takes complete responsibility for the integrity of the data and the accuracy of the data analysis.

## Data Availability

The data that support the findings of this study are available from the corresponding author upon reasonable request.
